# Psychometric properties and cultural adaptation of “LifeConScale” -Life Conditions Scale for Adolescents

**DOI:** 10.1371/journal.pone.0269644

**Published:** 2022-07-18

**Authors:** Beatriz Sánchez-Hernando, Ángel Gasch-Gallén, Isabel Antón-Solanas, Vicente Gea-Caballero, Javier Gállego-Diéguez, Juan Luis Sánchez-González, Iván Santolalla-Arnedo, Raúl Juárez-Vela, Ángela Durante

**Affiliations:** 1 Health Center “Amparo Poch”, Aragón Health Care System (SALUD), Zaragoza, Spain; 2 Nursing Research Group in Primary Care of Aragon (GIIS094-GENIAPA), Aragón Health Research Institute, Zaragoza, Spain; 3 Research Group in Primary Care of Aragon (GAIAP-GIIS011), Aragón Health Research Institute, Zaragoza, Spain; 4 Department of Physiatry and Nursing, University of Zaragoza, Zaragoza, Spain; 5 Faculty of Health Science, International University of Valencia, Valencia, Spain; 6 Research Group Blood Patient Management, IDIPAZ Madrid, Madrid, Spain; 7 Head of the Information, Transparency and Participation Service, Health Department, Government of Aragon, Zaragoza, Spain; 8 Department of Nursing and Physiotherapy, University of Salamanca, Salamanca, Spain; 9 Department of Nursing, University of La Rioja, Logroño, La Rioja, Spain; University of La Rioja, SPAIN

## Abstract

Promoting the adoption of healthy habits represents a great challenge for health and education professionals. In this sense, childhood and adolescence are propitious times for the acquisition and consolidation of behaviors and skills, being that numerous and different determinants act in the genesis of behavior. The purpose of this study was to test the Psychometric properties and cultural adaptation of “LifeConScale” -Life Conditions Scale for Adolescents-. A cross-sectional and multicenter study was carried out in a representative sample of adolescents enrolled in 1st and 2nd year of Compulsory Secondary Education in 18 educational centers in Aragon, during the 2018–2019 school year. Sociodemographic characteristics, life skills, daily habits, and academic performance were analyzed using an adapted questionnaire from different scales and previous studies. For the adaptation of the questionnaire, the expert panel technique was used and for its validation, exploratory factor analysis was carried out and Cronbach’s Alpha was applied, assessing the global internal consistency and of each one of the factors. The instrument showed a Kaiser-Meyer-Olkin sample size adequacy of 0.8122. A 6-dimensional model was chosen that explained 75.25% of the variance. The goodness of fit obtained a value of 0.802 in the Nomed Fix Index. The Comparative Fit Index was 0.891. The result of the analysis of variances and covariances carried out according to the Standardized Root Mean Square Residual yielded a value of 0.093 The analysis showed excellent application conditions in the study population and construct validity. This instrument will be useful for the evaluation of educational programs that work to promote health in educational centers, such as Health Promoting Schools.

## Introduction

Promoting the adoption of healthy habits represents a great challenge for health and education professionals. In this sense, childhood and adolescence are propitious times for the acquisition and consolidation of behaviors and skills, being that numerous and different determinants act in the genesis of behavior. The powerful influence that the school exerts on its students is beyond doubt and clearly demonstrated [1–3]. In fact, a good educational curriculum not only improves health outcomes but there are studies showing that when health is actively promoted at school, it can also improve student academic outcomes. There is evidence that school health promotion can support and add value to schools that aspire to achieve a set of social goals through their curriculum and a comprehensive school approach [2, 3]. Health promotion in the school environment is any activity carried out to improve or protect the health of all people who work, study, learn and live together in school. It is a broad concept that includes health education and also encompasses activities related to healthy school policies, the physical and social environment of the school, school methodology and programming, links with the community, and environmental services. In this sense, educational centers appear as a privileged space for the development of actions to improve health conditions, being a strategic sector for the realization of health promotion initiatives as a concept of "Health Promoting School", which encourages healthy human development and constructive and harmonious relationships. A Health Promoting School (HPS) has a comprehensive approach and aspires to improve the health and academic results of children and adolescents through learning and teaching experiences carried out at school, thus overcoming the strict and limited academic approach of educational institutions [1–5].

From this perspective, it develops factors linked to a participatory model of school organization with the creation of relationships and alliances in its environment, the performance of actions on the main determinants of health, incorporation of life skills, empowerment of basic skills, and their proper development [5–7]. In this context, we know that interventions in educational settings that incorporate HPS criteria in the long term are more effective than any type of specific activity that does not entail any continuity [4, 6]. In Aragon, each educational center can optionally be involved in different ways in the education and health promotion of the educational community. All centers include health content because they are integrated into the curriculum in a transversal way. At a second level are those centers that opt for a more intense treatment of certain health aspects. At a third level are the centers integrated into the Aragonese Network of Health Promoting Schools (ANHPS) [5]. The ANHPS, which began in 2008, has sought from its origin to be an innovative initiative and to introduce new ideas and approaches based on evidence and quality in school health promotion. This network has established an accreditation system, based on the factors developed by the educational centers, so that they are recognized as a Health Promoting School, which implies, on the one hand, transferring to the schools the values and pillars established in the Network Schools for Health as models of good practice, and secondly, to establish quality criteria that allow the experience to be evaluated and improved, both from the point of view of the center and that of the Network [3, 5].

Although the ANHPS carries out an annual evaluation of the implementation process of the program in the centers, so far, no evaluation of the results has been carried out. In addition, given the lack of scientific literature that analyzes the relationship between the Health Promoting Schools program with health outcomes and with students’ academic results, a project that evaluates the program in these terms is necessary, not only to be able to implement improvements in the program but to shed light on the reality of the educational sphere in our environment. To carry out the project with the maximum guarantees we need a tool that collects the information from all the fields that are worked on in the educational centers linked to the Network. Therefore, the objective of this study was to analyze the psychometric properties, of the “LifeConScale” instrument for adolescents enrolled in educational centers.

## Methods

### Study design and participants

A cross-sectional, multicenter design was used. Using a convenience sampling, during the 2018–2019 academic year a total of 1,047 first and second-year students of Compulsory Secondary Education were recruited, from 18 educational centers in Aragon that had to be enrolled in the academic year and present informed consent signed by a legal guardian. Those participants who did not understand the Spanish language were excluded. The participants completed a self-administered and anonymous questionnaire during the month of April 2019.

### Expert panel and study variables

The study variables were: sociodemographic characteristics (sex, academic year, age, number of siblings, position held among the siblings, people living together, father’s educational level, mother’s educational level, weight, height and health level perceived), life skills (cognitive skills, social skills and affect management), habits of daily life (diet, sleep, physical activity, use of screens and consumption of toxic substances) and academic performance.

The information was collected through an adapted questionnaire, from various sources: the Health Behavior in School-aged Children (HBSC) study [8], the General Self-Efficacy Scale [9], the first two subscales of the Social Skills Check List [10] and Affective Balance Scale [11]. For academic performance, we took the subjects corresponding to the courses selected according to the Department of Education of the Government of Aragon [12].

The instrument adaptation process, in which its viability was confirmed, was carried out using the expert panel technique [13]. The inclusion criteria to participate in the process were: a) being a nurse or doctor, b) experience in public health and / or community care (at least 5 years), c) with demonstrable participation in projects with Health Promoting Schools (at least 5 years). minus 5 editions). A total of 6 experts participated in two group sessions, each lasting approximately two hours, during the month of December 2017.

### Data analysis

A descriptive study of all the study variables was carried out to know the characteristics of the study population and the behavior of the different variables used.

For the validation of the instrument, an Exploratory Factor Analysis (EFA) of principal components was carried out to identify the dimensions in which the questionnaire was grouped. To ensure the applicability of the EFA in the study population, the Barlett Sphericity test was considered significant (p <0.05) and the Kaiser-Meyer-Olkin (KMO) measure of the adequacy of the sample size was greater than 0, 75 [14].

After carrying out this analysis, we included the factors with eigenvalues greater than 1 [15]. Cronbach’s Alpha was considered to evaluate the internal consistency of the factors and also the global consistency of the questionnaire.

With positive EFA results, a Confirmatory Factor Analysis (CFA) was performed, based on structural equation models. For the construction of the structural equation models, latent variables were created, calculated through the factors obtained in the previous section, and using the observed variables (items associated with each of the factors obtained). The goodness of fit analysis was performed with the following indices: chi-square (χ2), statistical probability (p), RMSEA (Root Mean Square Error of Approximation), CFI (Comparative Fit Index), SRMR (Standardized Root Mean Square Residual). An acceptable general fit corresponds to RMSEA <0.06, SRMR <1, and CFI> 0.90 [16, 17]. Excellent values correspond to CFI values greater than 0.95, RMSEA <0.05, and SRMR <0.08 [17, 18]. The SPSS v26 statistical package was used for the analyzes.

### Results

1047 students participated, with a mean age of 13.07 years (SD ± 0.82; range 12–16). More than half were girls. Most of the participants were in the 2nd year of Compulsory Secondary Education (CSE); more than 80% had at least one brother or sister and slightly less than 50% held the position of a younger brother. The vast majority of the participants lived with their father and mother. About a third of the fathers and mothers had vocational training. The mean weight was 51.87kg (SD ± 10.07), the mean height was 1.61m (SD ± 0.08). More than 90% of the participants considered that they were in good or excellent health (Table 1).

**Table 1:** Sociodemographic characteristics of the study population.

Variable	N (%)
Sex	
Boy	473 (45.26)
Girl	572 (54.74)
Course	
1st CSE	481 (45.94)
2nd CSE	566 (54.06)
Numbers of Brothers	
None	162 (15.47)
One	655 (62.56)
Two	164 (15.66)
Three	46 (4.39)
More than three	20 (1.91)
Position amor the brothers	
Higher	390 (44.42)
Intermediate	87 (9.91)
Less	401 (45.67)
Cohabiting	
Mother	1011 (96.75)
Father	863 (82.58)
Father’s partner	17 (1.63)
Mother´s partner	67 (6.41)
Grandmother	74 (7.08)
Grandfather	42 (4.02)
Foster Parents	1 (0.09)
Center of Minors	0
Other adult person	22 (2.11)
Brothers	472 (45.38)
Sisters	435 (41.94)
Father’s Education Level	
No studies	24 (2.45)
Primary	134 (13.67)
Secondary	275 (28.06)
Vocational Training	330 (33.67)
University	217 (22.14)
Mother´s Educational Level	
No studies	18 (1.80)
Primary	73 (7.31)
Secondary	254 (25.43)
Vocational Training	333 (33.33)
University	321 (32.13)
Health Level	
Excellent	402 (38.54)
Good	577 (55.32)
Tolerable	61 (5.85)
Bad	3 (0.29)

## Psychometric properties of the questionnaire

The instrument showed correct adequacy of the sample size (KMO = 0.8122) and Bartlett’s sphericity test with a p-value <0.001, which confirms its construct validity. As a solution to the exploratory factor analysis, 6 dimensions were selected, with an eigenvalue greater than unity, complying with Kaiser’s rule. Said 6-dimensional model explained 75.25% of the variance.

In Table 2, we show the variance that explains each of the defined factors, as well as its accumulated value. To analyze the internal consistency of the instrument, Cronbach’s alpha was used in each of the dimensions obtained in the factor analysis. The same table shows the values obtained for the factor, obtaining values that are high and greater than one, which indicates that each of the factors obtained through this analysis is consistent, the items that comprise it are stable in this dimension.

**Table 2:** Exploratory factor analysis.

Factor	Value	Difference	% Factor Variance	% Total Variance	Alpha de Cronbach
Factor1 Cognitive and social skills	7.58168	4.27937	31,64	31,64	0.8491
Factor2 Affective skills	3.30231	0.79993	13,78	45,42	0.7377
Factor3 Sleep	2.50238	0.39988	10,44	55,86	0.6369
Factor4 Nutrition	2.10250	0.76418	8,77	64,64	0.6503
Factor5 Use of Screens	1.33832	0.13412	5,58	70,22	0.7958
Factor6 Physical Exercise	1.20420	0.03601	5,03	75,25	0.6478
TOTAL	-	-	-	-	0.8465

Table 3 shows the items assigned to each factor, as well as the commonalities, found that show the degree to which the factors explain each variable. As can be seen, the communality values are generally high for all the items, which indicates that the 6 factors adequately represent the variables or questions of the questionnaire.

**Table 3:** Items assigned to each factor or dimension.

Item	Factor load item resulting from the EFA Commonality	Commonality
1: Cognitive and social skills		
I can find a way to get what I want, even if someone opposes me	0.2712	0.8903
I can solve difficult problems if I try hard enough	0.4389	0.7870
It is easy for me to persist in what I have proposed until I reach my goals	0.4438	0.7679
I am confident that I could effectively handle unexpected events	0.4776	0.6978
Thanks to my qualities and resources I can overcome unforeseen situations	0.5054	0.6523
When I am in difficulties I can remain calm because I have the necessary skills to handle difficult situations	0.4976	0.6364
Come what may, I’m usually able to handle it.	0.5476	0.6404
I can solve most problems if I try my best	0.4219	0.7624
If I find myself in a difficult situation, it usually occurs to me what to do	0.4832	0.7442
When faced with a problem, I usually come up with several alternatives on how to solve it.	0.5191	0.7203
Do you pay attention to the person who is speaking to you and make an effort to understand what they are saying to you?	0.4149	0.7607
Do you start a conversation with other people and then can you carry it on for a moment?	0.3370	0.7786
Do you talk to other people about things that interest you both?	0.2952	0.8128
Do you choose the information you need to know and ask the right person for it?	0.4738	0.6998
Do you tell others that you are grateful to them for something they did for you?	0.4182	0.7075
Do you make an effort to meet new people on your own initiative?	0.3221	0.7702
Do you introduce new people to others?	0.3481	0.7790
Do you tell others what you like about them or what they do?	0.3626	0.7848
Do you ask for help when you need it?	0.3549	0.7262
Do you join a group to participate in a certain activity?	0.3482	0.6306
Do you clearly explain to others how to do a specific task?	0.4898	0.7084
Do you pay attention to the instructions, ask for explanations and carry out the instructions correctly?	0.5022	0.6604
Do you apologize to others when you have done something that you know is wrong?	0.3010	0.8095
Are you trying to persuade others that your ideas are better and will be more useful than other people’s?	0.3592	0.9426
2. Affective skills		
Have you been bothered by someone?	0.4730	0.7451
Have you felt very lonely or distant from people?	0.5174	0.5840
Have you felt that things were going your way?	-0.3199	0.7502
Have you been very worried?	0.5858	0.6413
Have you been happy to have good friends?	0.2744	0.9054
Have you been afraid of what might happen?	0.6095	0.6254
Have you been particularly excited or interested in something?	0.3103	0.8366
Have you been feeling depressed or very unhappy?	0.5849	0.5803
Have you felt full of energy?	0.6187	0.5587
Have you felt very tired?	0.3427	0.8264
Have you been feeling very nervous, overwhelmed, or tense?	0.5640	0.6303
Have you felt like you were having a lot of fun?	0.7164	0.4777
Have you felt very happy or happy?	0.7020	0.4922
Have you ever felt like crying?	0.6416	0.5618
Have you felt euphoric (very happy or blissful)?	0.5832	0.6387
Have you felt secure about the future?	0.2641	0.8149
Have you been feeling bored?	0.2533	0.8634
Have you been happy or satisfied that you have achieved something?	0.5010	0.7019
3: Sleep		
How many hours do you usually sleep at night during the week?	-0.4538	0.7160
What time do you usually go to bed when you have school or institute the next day?	0.5053	0.6918
What time do you usually go to bed on weekends and during vacations?	0.5893	0.6254
4: Nutrition		
How often do you usually eat breakfast on the days that you have to go to school or institute?	0.3382	0.7968
How often do you usually eat breakfast on weekends?	0.2653	0.8663
How many times a week do you usually eat fruit?	0.2917	0.8498
How many times a week do you usually eat potatoes or salty snacks??	0.5148	0.6903
How many times a week do you usually eat vegetables or greens?	0.2938	0.8838
How many times a week do you usually eat sweets?	0.4965	0.7070
How many times a week do you usually drink soda or sugary drinks?	0.4927	0.7055
How many times a week do you usually eat meat?	0.2033	0.9085
How many times a week do you usually eat fish?	0.2065	0.9057
How many times a week do you usually eat or drink milk or dairy?	0.2636	0.8739
How many times a week do you usually eat cereals?	0.2965	0.8887
5: Use of Screens		
How many hours a day, in your free time, do you usually spend playing games on the computer, video console, Tablet, Smartphone or other electronic device (not including movement games or physical exercise)? Weekdays	0.6273	0.5705
How many hours a day, in your free time, do you usually spend playing games on the computer, video console, Tablet, Smartphone or other electronic device (not including movement games or physical exercise)? Weekend days	0.6610	0.5180
How many hours a day do you usually spend watching television, videos (YouTube or similar), movies, series and other entertainment on a screen? Weekdays	0.4248	0.6442
How many hours a day do you usually spend watching television, videos (YouTube or similar), movies, series and other entertainment on a screen? Weekend days	0.4457	0.6294
How many hours a day, in your free time, do you usually spend using electronic devices such as computers, tablets or smartphones to do homework, work, surf the Internet or social networks (Facebook, Twitter, Snapchat…)? Weekdays	0.4367	0.5422
How many hours a day, in your free time, do you usually spend using electronic devices such as computers, tablets or smartphones to do homework, work, surf the Internet or social networks (Facebook, Twitter, Snapchat…)? Weekend days	0.4263	0.5973
6: Physical exercise		
Outside of school hours. How often do you do some physical activity in your free time that makes you break into a sweat or short of breath?	-0.2000	0.9219
Outside of school hours. How many hours a week do you usually do any physical activity that causes you to break a sweat or short of breath during your free time?	0.4776	0.7119
Which of the following types of activities do you usually do in your free time? Physical activities as a team (soccer, basketball. . .)	0.4125	0.7677
Which of the following types of activities do you usually do in your free time? Individual physical activities (swimming, athletics, cycling. . .)	0.2728	0.8608
How do you usually go to school?	0.2042	0.9909

A CFA of the instrument was performed using structural equations. For the construction of the structural equation models, latent variables were created, calculated through the factors obtained in the previous section, and using the observed variables (items associated with each of the factors obtained). To analyze the goodness of fit of the model, the Nomed Fix Index (NFI) was calculated, obtaining a value of 0.802 and the RMSEA of 0.067. Furthermore, the CFI was 0.891. On the other hand, we calculated the variances and covariances of the study population and the existence of differences with the estimates obtained, from the SRMR, obtaining a value of 0.093 that indicates an excellent fit.

## Discussion

The present study focused on determining the psychometric characteristics after the cultural adaptation of the instrument "LifeConScale" (Life Conditions Scale for Adolescents) and verifying its usefulness to evaluate the results related to life skills, daily habits, and academic performance of adolescents in school. The analysis showed excellent conditions of application in the study population and construct validity, surpassing the results of other questionnaires that had previously been partially applied, following the theory that underlies this topic. This is the example of the HBSC Spain 2014 study [8] for which no data has been found on the validation of its questionnaire. In the case of the General Self-efficacy Scale and the Goldstein Social Skills Scale, they also had good data on internal consistency and validity [9, 19]. This is not the case with the scale that measures effective balance [11], which yields more modest data for internal consistency. Six factors emerged that grouped the study variables on life skills and daily habits: cognitive and social skills, an effective balance, sleep, diet, use of screens, and physical exercise.

In four of the 6 factors we observed, cognitive and social skills were grouped in the same dimension so that unlike other studies [9, 20–22], it seems a more valid construct than the questions related to these two types of ability are carried out jointly rather than separately. Following this line of argument, different daily habits such as sleep, diet, use of screens, and physical activity were grouped, improving the adolescent’s global vision and offering more information than other studies that focus on one of the dimensions.

Comparing the result of the validation of the different factors of our tool with those of other studies, we find that in factor 1 "Cognitive and social skills" the internal consistency is 0.8491, compared to that found in the General Scale of Self-efficacy of 0.89 [9] and that of the Goldstein Social Skills Scale 0.905 [19]. Regarding factor 2 "Affective skills", a Cronbach’s alpha value of 0.7377 was obtained, compared to the Affective Balance Scale, which obtained a score of 0.47 [11]. The rest of the factors we do not have an equivalence with which to compare, however, their adjustments are good.

To date, there are no studies that have performed validation of an instrument that brings together these parts. This instrument will be useful for the evaluation of educational programs that promote health in schools, such as HPS. This tool allows obtaining quality indicators since it offers information on each of the key points described in the EPS program [5]. In our specific case the ANHPS allows the evaluation of the results of the program and the detection of changes before and after its implementation.

### Limitations

Regarding the validation method, with the analyzed models and the goodness of fit, the Normed Fit Index has limitations since it depends on the number of parameters to be estimated, which in our case is high due to the number of items associated with the instrument. The Normed Fit Index penalizes the quality of fit of the model with the number of estimated coefficients necessary to achieve the level of fit and therefore is not a good measure of goodness of fit.

## Conclusion

Our study has shown that the psychometric properties and the cultural adaptation to Spanish population has a factorial validity and could be used in clinical practice and research to measure evaluation of educational programs that work to promote health in educational centers, such as Health Promoting Schools.

## Supporting information

S1 Data (XLSX)
